# Multicentric primary ectopic meningiomas involving lung and cranial bone barrier: A rare case report

**DOI:** 10.1097/MD.0000000000041294

**Published:** 2025-01-31

**Authors:** Huiyang Zhang, Xiang Li, Yan Jiang

**Affiliations:** a Department of Radiology, The Central Hospital of Wuhan, Tongji Medical College, Huazhong University of Science and Technology, Wuhan, P.R. China.

**Keywords:** multiple pulmonary nodules, primary intraosseous meningioma, primary pulmonary meningioma

## Abstract

**Rationale:**

Primary ectopic meningiomas (PEMs) are extremely rare, with limited literature available on the subject. Understanding their clinical and radiological characteristics is crucial for accurate diagnosis and treatment.

**Patient concerns:**

This study presents a case of a multicentric PEM involving the lungs and cranial bone barrier, aiming to provide new insights into its clinical and diagnostic features.

**Diagnoses:**

A 46-year-old woman with no prior history of intracranial meningiomas or other tumors was found to have multiple lobulated nodules in the lungs during a routine physical examination. Computed tomography scans revealed well-defined lesions with mild to moderate heterogeneous enhancement. Magnetic resonance imaging showed a lesion at the cranial bone barrier, which presented as a high-signal area on T2-weighted FLAIR images and mild to moderate enhancement on T1-weighted images. The magnetic resonance spectroscopy displayed a broad Lip peak but lacked N-acetylaspartate or creatine peaks. Histopathological and immunohistochemical analyses confirmed the diagnosis of multicentric PEMs.

**Interventions:**

We performed surgical resection of the lesion on the cranial bone plate and conducted follow-up examinations for the multiple lesions in the lungs.

**Outcomes:**

This case highlights the diagnostic challenges of multicentric PEMs involving the lungs and cranial bone barrier. Due to their extremely low incidence and nonspecific clinical manifestations, a comprehensive evaluation combining radiological, pathological, and immunophenotypic data is essential for accurate diagnosis.

**Lessons:**

This case underscores the importance of a thorough, multidisciplinary approach to diagnosis and treatment and provides valuable insights for managing similar rare cases.

## Graphical abstract

**Figure:**
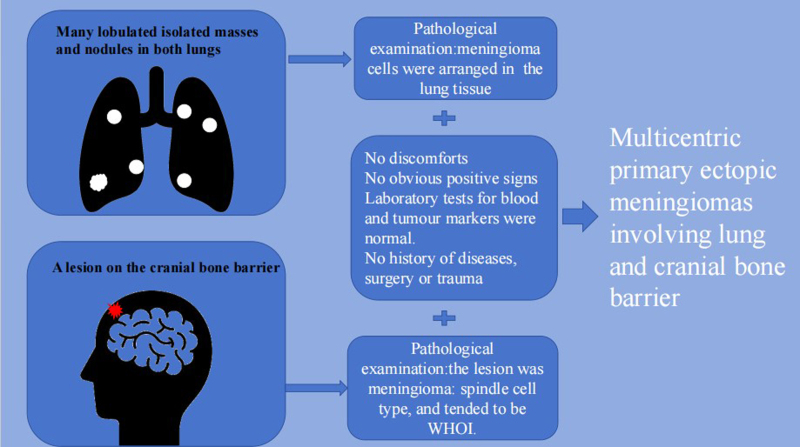

## 1. Introduction

Meningiomas are the most prevalent type of tumor in the central nervous system, representing approximately 36.1% of all central nervous system tumors and an incidence rate of 7.61/100,000,^[1]^ These tumors originate from the arachnoid meningeal epithelium and constitute 15% to 20% of intracranial tumors.

Primary Ectopic Meningioma refers to meningiomas occurring outside the central nervous system, broadly speaking, it refers to tumors occurring in tissues and organs not covered by the meninges under normal conditions, which are not related to normal meningiomas and have the morphological and structural characteristics of meningiomas, which are relatively rare in clinic, accounting for only 1% to 2%.^[2]^ Primary ectopic meningiomas are rarely reported in domestic and international literature. Primary intraosseous meningioma refers to meningiomas occurring in the epidural bone,^[1]^ the vast majority of which occur in the skull, mostly in women, and cranial bone barrier-type primary meningioma is one of them. Primary pulmonary meningioma (PPM) has been reported in domestic and abroad since 1982 when Kemnitz et al.^[3]^ Recently, a patient with pulmonary and cranial plate barrier type primary meningioma was seen in our department, and the imaging features, diagnosis and differential diagnosis of this tumor are discussed, which will provide some support for the early and accurate diagnosis of this disease.

## 2. Case report

A 46-year-old women was referred to our hospital because of pulmonary occupations found during medical checkup. The patient had no obvious cough, sputum, wheezing, fever, chills, chest tightness, chest pain, hemoptysis, night sweats and other discomforts. There was no history of hypertension, coronary heart disease, diabetes mellitus, respiratory diseases, or history of surgery or trauma. The patient was physically fit, with no history of smoking or alcohol consumption, and no special medical history in the family. Physical examination: clear respiratory sounds in both lungs, no dry or wet rhonchi, flat and soft abdomen, no pressure or rebound pain, no obvious percussion pain in both renal regions, and no obvious positive signs. Laboratory tests for blood and tumor markers were normal.

**Figure 1.**
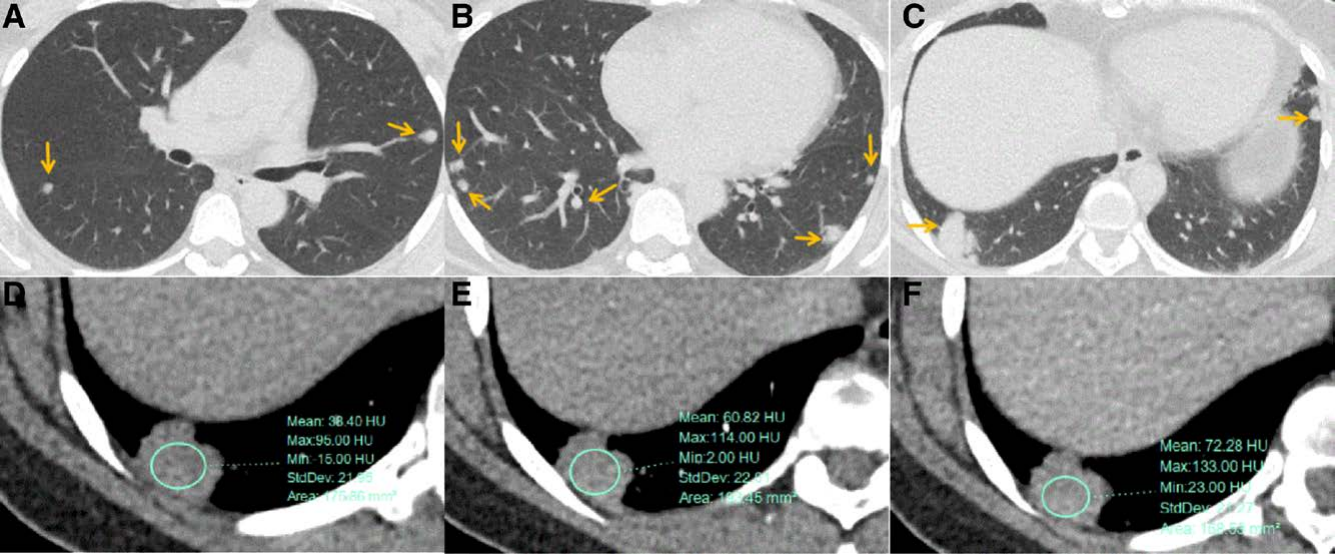
(A–C) Chest CT images showing multiple nodules in both lungs, the largest one is located in the right lower lung; (D–F) Enhancement scans of the largest lesion showing mild to moderate enhancement, which is less homogeneous. CT = computerized tomography.

**Figure 2.**
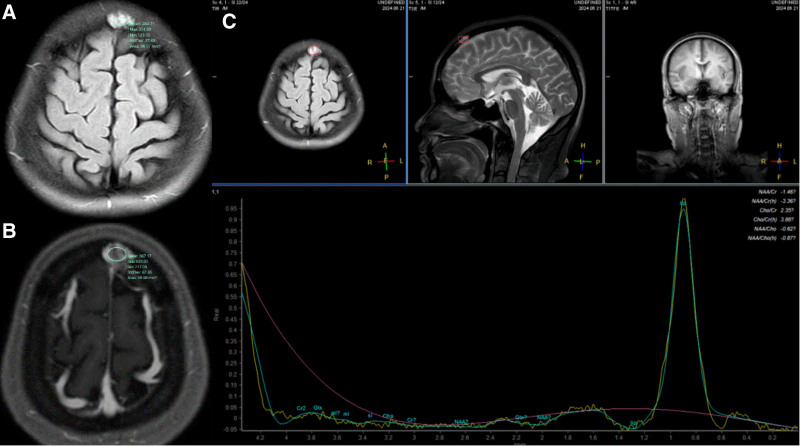
(A and B) The lesion in the cranial bone barrier shows a high signal in T2WI Flair images, and mild to moderate enhancement in T1-weighted MR image; (C) The MRS image no show NAA peak and Cr peak of the lesion, and show the broad Lip peak.

**Figure 3.**
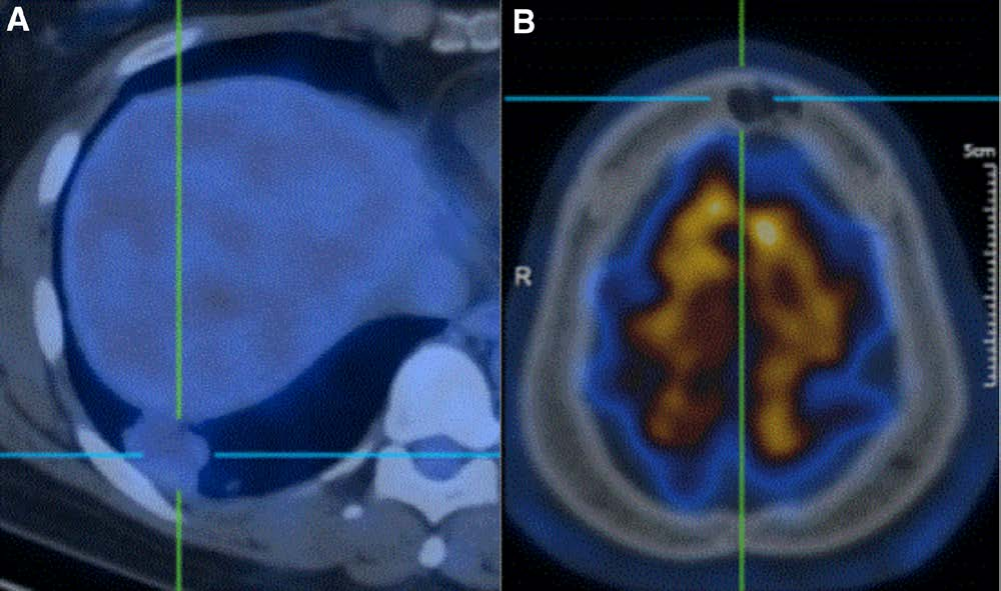
(A) Mildly increased metabolism nodules on PET/CT in the right lower lung; (B) Mildly increased metabolism lesion on PET/CT in the cranial bone barrier. CT = computerized tomography.

Imaging examination: cardiac, abdominal and urinary ultrasound medical examinations showed no obvious abnormality. Contrast-enhanced chest computerized tomography (CT) revealed several isolated nodules on both lungs with well-defined lobes (Fig. 1A–C), the largest one where was located in the right lower lobe showed mild to moderate heterogeneous enhancement (Fig. 1D-F). MRI of the lesion in the cranial bone barrier showed a high signal in T2WI Flair images, and mild to moderate enhancement in T1-weighted MR images; the MRS images no show NAA peak and Cr peak, and show the broad Lip peak (Fig. 2A–C). Multiple pulmonary nodules and the lesion in head had mild fluorodeoxyglucose (FDG) uptake (the largest nodule with a SUVmax of 2.5, other nodules with a SUVmax of 1.0 to 1.2, the lesion in head with a SUVmax of 2.1) on 18 F-FDG PET/CT (Fig. 3A and B).

**Figure 4.**
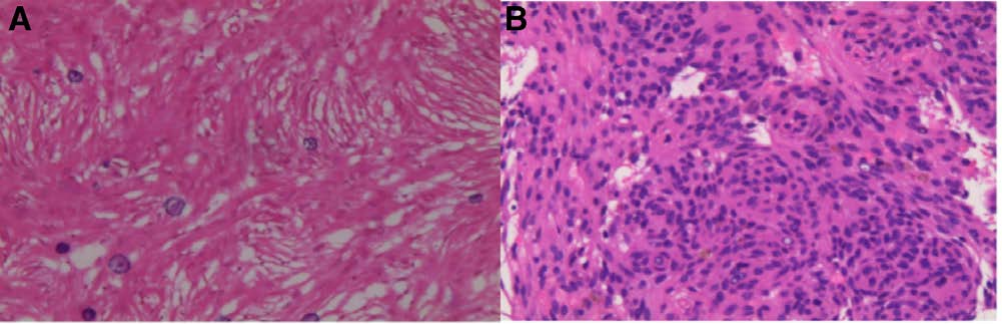
(A) The boundary of lesion in the cranial bone barrier is clear, the cytoplasm is eosinophilic, and the nucleus is ovoid; (B) The right lower lung nodule puncture microscopy observed that the meningioma cells in the lung tissue were arranged in the form of a whirlpool; the nucleus was large, the nuclear membrane was clear, and the nucleolus could be seen.

Pathological examination: The lesion under the cranial bone barrier was described as a piece of grayish-white tissue of 1.5 × 1.5 × 0.5 cm in size, hard as a stone, and microscopically the spindle cell lesion within the cranial plate baffle was examined and detected as having a still-clear border, eosinophilic cytoplasm, and ovoid nuclei (Fig. 4A). The Immunohistochemistry results were: Vimentin (+), PGP9.5 (+), S-100 (+), EMA (+), SSTR2 (+), SMA (+), E-cad (a few +), PR (individually +), P53 (−), CD34 (−), STAT-6 (−), SOX-10 (−), and Ki-67Li about 1%, which was in line with the meningioma: spindle cell type, and tended to be WHO grade I. Two linear samples of 0.8 to 1.0 cm in length and 0.1 cm in diameter were taken from the right lower lung nodule, and under the microscope, we saw meningioma cells were arranged in a swirling pattern in the lung tissue; the nucleus was large, the nuclear membrane was clear, and the nucleolus could be seen (Fig. 4b). The Immunohistochemistry results were: Vimentin (+), E-cad (+), EMA (+), CD56 (+), CD34 (+), PGP9.5 (+), S-100 (partly +), PR (partly weakly +), STAT-6 (partly plasma cell +), CK7 (−), TTF-1 (−), NapsinA (−), P40 (−), CK5/6 (−), GFAP (−), CgA (−), Syn (−), Pan-CK (−). Ki-67Li was about 2%, and was considered to be a primary pulmonary meningioma.

## 3. Discussion

Primary ectopic meningioma occurs at different sites and varies in size, morphology and imaging manifestations. Ectopic meningiomas are commonly found in the head and neck, especially in the scalp, skull, brain parenchyma, orbits, nose, paranasal sinuses, middle ear, parotid glands, oral cavity, inferior colliculus, and also in the bones, lungs, mediastinum, peripheral nerves, skin, and peritoneum, and are usually located in the vicinity of the mid-axis of the body.^[4–7]^ The pathological types of ectopic meningiomas are the same as intracranial meningiomas, which are classified into: epithelial cell type, fibroblast type, vascular type, gravel body type, and osteochondrocyte type, with the former two being more common. Most of the primary ectopic meningiomas are characterized by painless enlarged masses, which are often found during physical examination. The clinical manifestation of this disease is often nonspecific, which mainly depends on the site of tumor occurrence, size and the degree of invasion and destruction of the surrounding tissues, so it is easy to be misdiagnosed in clinic.

PPM is relatively rare, with more than 60 cases reported in the literature both in demotic and abroad since its first discovery in 1982. The tumor is generally benign, slow-growing, often with no specific clinical symptoms. The easiest imaging method for PPM is X-ray, which can show a spherical lesion, while CT and MRI can help to determine the extent, size, contour, density, calcification and necrosis of the lesion.^[5]^ The most common presentation is isolated, round, solid, well-defined pulmonary nodules and masses, with or without calcification, ranging in size from 0.6 cm to 6 cm.^[6,7]^ In this case, the mass may have heterogeneous enhancement, which is different from the uniform enhancement of primary meningioma.^[8]^ However, unlike the previously reported cases, this case showed multiple pulmonary nodules with clear borders on chest CT, and the larger one was lobulated, and there were no special signs such as calcification, solid changes, cavities, etc., which was similar to the manifestation of pulmonary metastasis. Because the pathological mechanism of distant metastasis of ectopic meningioma has not been developed, we considered this case as a multicentric primary pulmonary ectopic meningioma, and there are also cases that reported that primary pulmonary meningiomas can present as multiple nodules in both lungs.^[9]^ On 18F-FDG PET, PPM exhibited mild to high metabolic activity with a mean SUVmax of 4.36 and a range of 0.6 to 12.9.^[10]^ In this case, the majority of the multiple nodules in both lungs showed mild abnormal concentrations of the contrast agent, with a SUVmax of 1.0 to 2.5. The most important immunohistochemical markers that support the diagnosis of PPM are EMA (+) and Vimentin (+), and other markers such as CK, S-100 protein, and CD34 may be focally positive.^[9]^ Intracranial meningiomas can also metastasize to the lungs, but it is inconclusive whether ectopic meningiomas can develop pulmonary metastases, so a thorough patient history should be taken before diagnosing a primary meningioma.

The primary intraosseous meningioma in our patient was the cranial plagiocephaly type, and the clinical manifestations of cranial plagiocephaly meningioma are mostly limited elevated, progressively enlarged bony masses in the brain, with occasional symptoms of headache, and the patient in this case had no positive signs and symptoms.^[9]^ Cranial plagiocephaly meningiomas account for about 1% to 2% of all meningiomas and 66.7% of epidural meningiomas.^[11]^ Cranial plagiocephaly meningiomas have no specific imaging manifestations, and can be manifested as osteomalacia and osteolytic bone destruction, which is very easy to be misdiagnosed as a primary bone tumor, with the former being more common, and the rare cases showing mixed bone destruction.^[12]^ Osteogenic bone destruction on CT shows dense and thickened bone, or glass-like change, and the boundary between inner and outer plates is not clear.^[3,8]^ In the case of osteoblastic tumor, the boundary between the inner and outer plates is not clear, and the edge may be irregularly jagged or lace-like, and arc-shaped calcification is occasionally seen in the adjacent dura mater, which indicates meningeal infiltration, and no obvious enhancement. Due to the dense and sclerotic bone of osteogenic tumor, the T1WI and T2WI mostly show low signal, which is poorly displayed. Osteolytic destruction is mostly manifested as bone destruction and soft tissue mass in CT, and the inner and outer plates of the skull can be destroyed and disappeared or partially survived, and the mass can be enhanced with more uniform and obvious enhancement in CT; the MRI show equal, low T1WI, and equal, highT2WI, and the enhancement is obvious and even. In this case, MRI of the subplaque tumor showed low signal in T1WI, high signal in T2WI, and obvious and uniform enhancement. The pathology of subplaque meningioma was mainly epithelial, followed by fibroblastic type, and clear cell type was rare. Immunohistochemistry was similar to that of intracranial meningiomas, and most of them were Vimentin (+) and EMA (+), which were generally regarded as benign tumors, but clear meningiomas are WHO II, which are invasive, and have a high probability of recurrence.^[7]^ Butscheidt et al^[4]^ showed that 91% of intraosseous meningiomas were WHO I, as was the case here.

In this case, the lesion was found incidentally during physical examination without corresponding clinical symptoms, and the imaging and pathological examination confirmed that the cranial platysma barrier and the lung lesion were meningiomas. On the premise that there was no definite meningioma in the cranium, and in combination with the available examination data, it was more inclined to be meningiomas with a multicentre ectopic origin, but it was not possible to confirm the existence of a relationship between multiple nodules in the cranium and the lungs at this point in time. There would be several views: the platysmal meningioma and the right lower lung meningioma are both primary ectopic and the relatively small nodule in the lung is a metastasis of the large nodule of the most proven meningioma in the right lower lung, which is caused by the proliferation of the ectopic arachnoid cells in the lung; the platysmal meningioma and all the nodules in the lungs are primary ectopic meningiomas; and the cranial platysmal meningioma causes multiple metastases in the lungs. Surgical total resection is the treatment of choice for cranial plagiocephaly meningiomas, and the postoperative prognosis is good. Thoracoscopic minimally invasive surgery is preferred for PPM to preserve as much lung tissue as possible and to improve the patients’ quality of life after surgery.

Differential diagnosis: CT of PPM should be differentiated from intrapulmonary lesions such as misshapen tumor, inflammatory pseudotumour, primary peripheral lung carcinoma and sclerosing haemangioma: misshapen tumor nodules are characterized by calcification and fat density, especially popcorn-like calcification. Inflammatory pseudotumour nodules have clear borders and are characterized by nodules with knife-cut margins, which tend to be mildly enhancement on CT-enhanced scans. Primary peripheral lung cancer nodules are often accompanied by lobulation, burr, pleural concavity sign and vascular aggregation sign, and may be accompanied by mediastinal and hilar lymph node enlargement, with mild to moderate enhancement. Sclerosing haemangioma nodules develop a clear interface sign and show significant homogeneous enhancement with vascular adjacency on enhancement. Cranial plagiocephaly meningioma needs to be differentiated from cranial osteoma, abnormal proliferative foci of bone fiber, cranial eosinophilic granuloma, metastasis, etc. Cranial osteoma bone often manifests itself as a dense elevation of the inner and outer plates of the skull with a wide base and smooth edges. Abnormal bone fiber proliferation syndrome cranial bone progressive expansion, diffuse expansion and thickening of bone. Eosinophilic granuloma of the skull is often associated with local soft tissue swelling. Bone metastases tend to present as multiple lesions with a history of primary disease. The existing diagnostic criteria for primary ectopic meningiomas are: occur in an anatomical site without meningeal tissue, not be associated with intracranial or intraspinal meningiomas, and must have the histological structure of a typical meningioma.^[4,5,9,13]^ The combination of laboratory tests and imaging of other parts of the body is beneficial to the differential diagnosis, but the final diagnosis still requires pathological examination.

The study have some limitations: The imaging examinations of the patient are not comprehensive. Such as MRI functional sequences, including DWI, PWI, DTI, and fMRI, as well as CT perfusion imaging, SPECT and DSA can provide significant diagnostic value for this disease. While the report discusses potential mechanisms of multicentric ectopic meningiomas, there is insufficient experimental or genetic or molecular studies to support these hypotheses. Due to the rarity of this case, the study focuses on a single patient, and significant differences in the diagnosis of such diseases may exist across different institutions.

## 4. Conclusion

This study presents a rare case of multicentric primary ectopic meningiomas involving both the lungs and cranial bone barrier. The patient, who had no obvious clinical symptoms, was incidentally found to have multiple pulmonary nodules during a routine physical examination. Further brain MRI and PET-CT scans revealed additional lesions in the cranial bone barrier. Through a combination of imaging, histological analysis, and immunohistochemistry, the pulmonary nodules and cranial bone barrier lesions were identified as meningioma cells. The study systematically ruled out other potential diagnoses, including primary meningiomas, metastatic tumors, primary bone tumors, and inflammatory pseudotumors, leading to a definitive diagnosis.

In conclusion, diagnosing multicentric primary ectopic meningiomas requires the exclusion of extrameningeal spread or metastasis from intracranial meningiomas. A comprehensive diagnostic strategy, incorporating multiple imaging modalities, is essential. When lesions involve the cranial bone barrier, brain CT or MRI, with various functional sequences such as contrast enhancement, DWI, PWI, and DTI, can aid in diagnosis. For pulmonary lesions, routine chest CT with contrast, chest MRI, and PET-CT scans are critical in evaluating the extent of the disease and excluding involvement of other sites, facilitating a more accurate and holistic diagnosis. Given the nonspecific clinical manifestations of most ectopic meningiomas, these lesions are often mistaken for other benign or malignant tumors, complicating the diagnostic process. Therefore, in addition to imaging and laboratory tests, biopsy may be necessary to obtain histopathological confirmation for definitive diagnosis. This case underscores the importance of considering rare entities such as multicentric primary ectopic meningiomas when encountering multicentric lesions in clinical practice. While metastatic tumors are frequently suspected, it is crucial to also entertain the possibility of less common and even rare conditions, like multicentric primary ectopic meningiomas.

## Author contributions

**Investigation:** Yan Jiang.

**Writing – original draft:** Huiyang Zhang.

**Writing – review & editing:** Xiang Li.
